# *Infectious Diseases of Poverty*: 10 years’ commitment to One Health

**DOI:** 10.1186/s40249-021-00914-4

**Published:** 2021-11-03

**Authors:** Xiao-Nong Zhou

**Affiliations:** 1 National Institute of Parasitic Diseases at China CDC/Chinese Center for Tropical Diseases Research, WHO Collaborating Centre for Tropical Diseases, NHC Key Laboratory for Parasite and Vector Biology, Shanghai, 200025 People’s Republic of China; 2 School of Global Health, Chinese Center for Tropical Diseases Research, Shanghai Jiao Tong University School of Medicine; One Health Center, Shanghai Jiao Tong University-The University of Edinburgh, Shanghai, 200025 People’s Republic of China

November 3, 2021 marks the sixth annual One Health Day, a global event promoting the One Health approach to solve problems at the human-animal-environment interface. It provides an opportunity to encourage all stakeholders, including physicians, veterinarians, pet owners, policymakers, disease detectives, people in the laboratory, farmers, law enforcement staff and others to work together towards improving health for people, animals, plants and their shared environment. The *Infectious Diseases of Poverty* is an open-access journal calling for more research in line with the One Health concept. This was enacted at the very start of the journal in October 2012 [1–3], e.g., the inaugurated article of the journal recommended “One Health-One World” as a way to set the stage for publishing research results strategically focused on this approach [4]. Based on recommendations by the UNICEF/UNDP/World Bank/WHO Special Programme for Research and Training in Tropical Diseases, *Infectious Diseases of Poverty* encouraged funding on infectious diseases be particularly awarded to scientists who adopted a collaborative, multisectoral and transdisciplinary approach with a focus on public health and the human-animal-environment interface. In the following years, the status of adverse global change of climate and environment was reviewed with special reference to the journal’s role in developments conducive to initiation of pandemics now and in the future. Several articles were published discussing the high burden of disease arising via anti-microbial and insecticidal resistance, malnutrition, conflict and other occurrences where the public health approach showed signs of strain [5]. Early on, the journal called attention to issues of public health importance, especially paying attention to existing well-known and infectious diseases of poverty and the changing milieu caused by today’s changing environment, stressed life and determinants indicating the risk for eroding public health activities amid ailing civilization-supporting systems [6].

The original goal of *Infectious Diseases of Poverty* was to address essential public health questions relating to infectious diseases of poverty and provide a platform to communicate with all partners promoting the One Health approach. This is still the case, and the journal continues to set research priorities supporting the control of infectious diseases of poverty through publishing original and empirical work on transdisciplinary research at the local, regional, national and global levels. As the founding Editor-in-Chief, I am very proud that the *Infectious Diseases of Poverty* has not only achieved its previously set goals by being an advocacy platform for the translation of new knowledge into policy and by the promotion of large-scale programmes to combat infectious diseases of poverty, but that it has also facilitated the much needed dialogue between policy makers, public health practitioners, control staff and academic researchers and their donors.

**Fig. 1:**
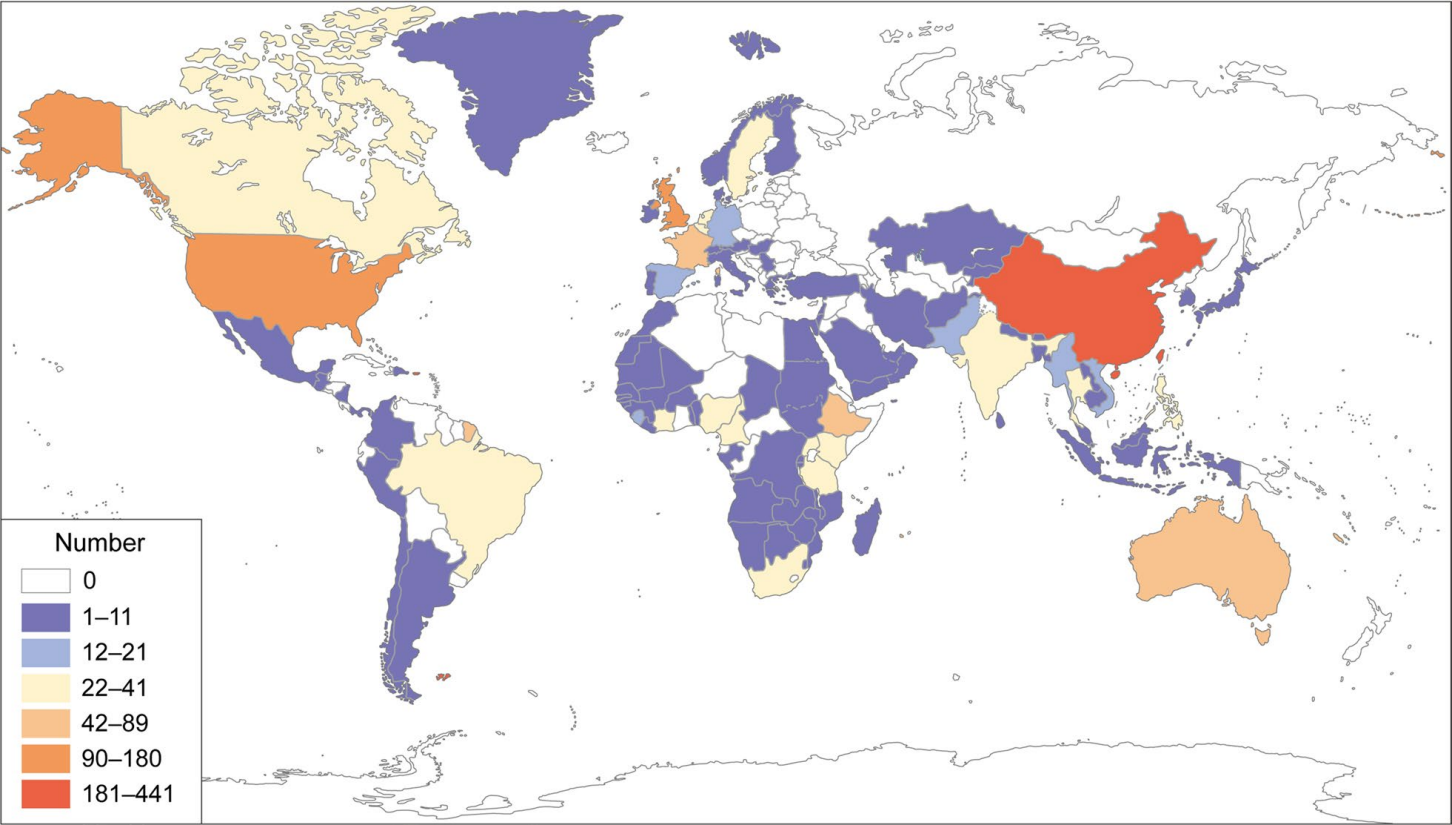
The map of the countries/regions (n = 105) where authors of all publications came from

**Fig. 2:**
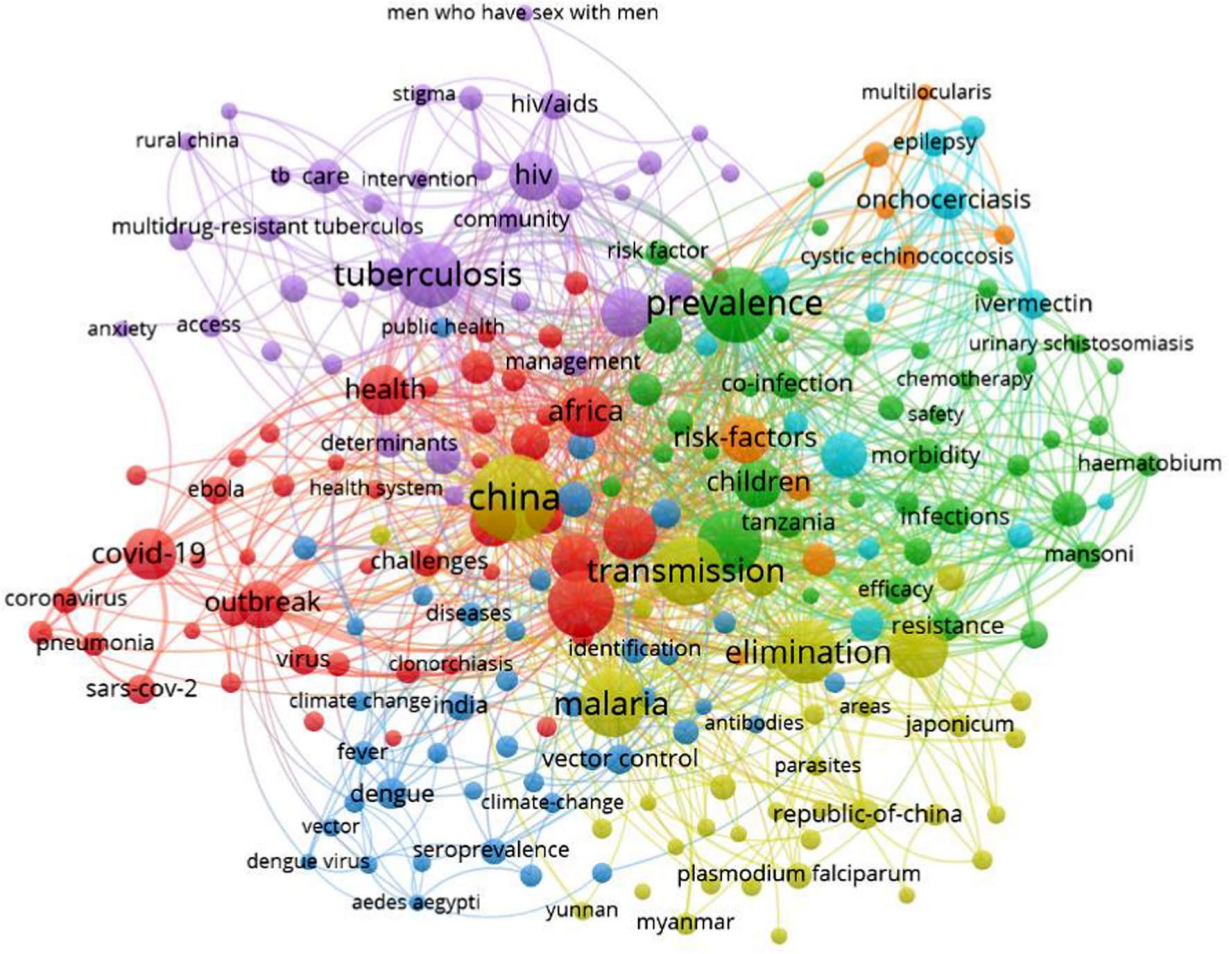
The cloud map of the areas and disease spectrum covered by articles published in *Infectious Diseases of Poverty* since its inauguration in 2012

In relation to knowledge translation, *Infectious Diseases of Poverty* has published 949 articles selected from more than 3308 submitted papers originating in 120 different countries as of October 10, 2021 since its inauguration in 2012. Those publications cover 105 countries/regions prepared by 4785 authors and co-authors from 3392 academic institutions (Fig. 1). Results from an analysis of the cloud computing by containing all those publications show that the focus has been on infectious diseases of poverty, such as tuberculosis, malaria, schistosomiasis, COVID-19, Ebola, dengue, onchocerciasis, Chagas disease, brucellosis, echinococcosis, epilepsy and other vector-borne diseases. Research on these diseases and many others involved a large number of disciplines, e.g., epidemiology, prevalence surveillance, outbreak response, mitigation of climate changes, drug resistance, insecticide resistance, vector control, disease elimination, spatial analysis, co-infections and syndemics, health education, ecological management, also including multi-disciplinary approaches (Fig. 2). Dealing with important diseases and endemic areas with the specific options mentioned has made the journal well recognized by the community of researchers and control staff working in the field of infectious diseases, as exemplified by more than 2.2 million downloads and a high citation rate resulting in a Science Citation Index impact factor for 2021 as high as 4.52. Based on the Journal Citation Report (2021), *Infectious Diseases of Poverty* ranked as the top journal among 23 journals in the category of tropical medicine, as the fourth top journal among 38 journals in the category of parasitology and as the 27th among 92 journals in the category of infectious diseases.

In relation to dialogue and communication, our editorial board members consisting of 58 outstanding scientists from 25 countries, contributed significantly to organizing thematic series, inviting review articles, formulating scoping review guidelines, coordinating forums/workshops, etc. For example, more than 300 participants attended six workshops on scientific writing to improve their capacity to report on control of infectious diseases of poverty, and the audience at the digital forum, *i.e.*, IDEA Forum—lessons from authors, exceeded 3600 participants from the world learning on how to select research topics and write better scientific papers, through the interview among readers, authors and editors. In addition, more than 600 instances of activities to advocate methodologies, e.g., new media approaches, were held to reach out and engage more scientists and partners in research and publication on infectious diseases of poverty. A majority of authors involved came from the developing world and diseases endemic countries, including Brazil, Cameroon, Ethiopia, India, Islamic Republic of Iran, Nigeria, Malaysia, Pakistan, South Africa, Thailand and United Republic of Tanzania, which speaks to the unique character and success of the forum.

The optimal goal of *Infectious Diseases of Poverty* is to reduce the disease burden through promoting the interconnection between people, animals, plants and their shared environment. As an international journal with a view to focus on the broadness and interface of the One Health approach, we will continue to engage in increased activities to improve communication, coordination and collaboration among partners in accessing relevant public health interventions efficiently, identifying potential gaps for further research and public health actions, and providing scientific evidence for policymaking and policy translation. For example, beside the current types of articles we published, such as research article, scoping reviews, case report, case study, short report, commentaries, letters to the editor and opinion, we will next year launch policy briefs, i.e. entries related to One Health governance which is still a neglected area with respect to implementation for One Health. It was decided to engage more in communication, collaboration and coordination with partners whose interests across the spectrum of human, animal and environmental sectors. Through the policy briefs' articles, we expect to publish more articles on “hot topics” in the area of zoonosis intervention, microbial resistance control, food security and health impacts of climate change.

Far from resting on its laurels, *Infectious Diseases of Poverty* celebrates the first 10 years of publication by continuing to shoot for the stars. In the last ten years, the journal has strongly focused on promoting trans-disciplinary research bridging research and policy, which has taken the form of scoping reviews providing comprehensive and authoritative coverage of a topic area with a fast peer review for submitted papers. Although *Infectious Diseases of Poverty* was awarded the mark of Editorial Excellence by Springer Nature for its highly rated publishing record in 2020, the editorial office will make further efforts to better serve authors and reviewers by shortening the article processing time and attempt to further improve the quality of article.
